# South African speech-language therapists’ perceived competencies and use of Makaton

**DOI:** 10.4102/sajcd.v73i1.1154

**Published:** 2026-02-25

**Authors:** Chriséle Mc Geer, Carmen Milton, Carlien Vorster, Marien Alet Graham

**Affiliations:** 1 Department of Speech-Language Pathology and Audiology, Faculty of Humanities, University of Pretoria, Pretoria, South Africa; 2 Department of Mathematics Education, College of Education, University of South Africa, Pretoria, South Africa

**Keywords:** augmentative and alternative communication, unaided, Makaton, communication disorders, speech-language therapists, South Africa

## Abstract

**Background:**

Makaton, an unaided augmentative and alternative communication (AAC) method, is widely used by speech-language therapists (SLTs) in diverse clinical and educational settings. Despite its applicability across populations with complex communication needs, limited research has explored how South African SLTs perceive, use and experience Makaton. This gap is significant given South Africa’s multilingual context, resource disparities and the need for culturally relevant AAC strategies.

**Objectives:**

This study aimed to explore South African SLTs’ perceived competencies and use of Makaton.

**Method:**

This study employed a mixed-methods design. An online survey comprising closed- and open-ended questions was distributed to SLTs across South Africa. A total of 57 participants were included in the study. Quantitative responses were analysed using descriptive and inferential statistics, while qualitative responses were analysed thematically using Braun and Clarke’s six-phase framework.

**Results:**

While 87.7% of participants viewed Makaton as valuable, only 5.5% reported feeling very confident using it. No statistically significant difference between recommending Makaton to parents and team members (Wilcoxon signed-rank [WSR] = −1.386, *p* = 0.166). Four themes captured Makaton’s perceived value: (1) multimodal communication, (2) accessibility and practicality, (3) support for speech and language development and (4) inclusion and social interaction. Reported challenges included: (1) limited awareness and training, (2) financial and/or resource constraints, (3) poor consistency and carryover, (4) motor and/or cognitive limitations and (5) cultural and regional mismatches.

**Conclusion:**

While Makaton is positively regarded by SLTs, limited training, confidence and implementation support hinder consistent use.

**Contribution:**

Expanding access to training and embedding Makaton in professional education may enhance AAC service delivery in the multilingual South African context.

## Introduction

Augmentative and alternative communication (AAC) is an umbrella term used to describe methods to support and supplement communication for individuals with complex communication needs (Iacono et al., 2016). It can be aided (e.g. picture communication boards or speech-generating devices) and/or unaided (e.g. signs, gestures or facial expressions) (Ganz et al., 2014; Handberg & Voss, 2018; Iacono et al., 2016; Pattison & Robertson, 2016). It is used in a wide variety of settings, including educational, vocational and social environments (Handberg & Voss, 2018; McNaughton et al., 2019). It can foster greater independence and community participation and also empower individuals to advocate for themselves, leading to more fulfilling lives (Tönsing & Dada, 2016; Waddington et al., 2017).

One multimodal, unaided AAC method, widely used by speech-language therapists (SLTs) internationally, is Makaton (Faiz et al., 2022). Makaton integrates signs, symbols and spoken language to support communication for individuals with communication difficulties, including those with intellectual disabilities and neurodevelopmental disorders such as Autism Spectrum Disorder (ASD) (Makaton South Africa, n.d.). It is a total communication approach (Faiz et al., 2022) offering visual and physical means to facilitate improved understanding, expression of needs and desires, and social interaction for individuals with complex communication needs (Bednarski, 2016; Brignell et al., 2018; Faiz et al., 2022). Research indicates that Makaton supports the development of both language comprehension and expression (Bednarski, 2016; Faiz et al., 2022) and may also promote literacy in individuals with severe learning difficulties (Mistry & Barnes, 2013). Signs, one facet of Makaton, provide an alternative communication method that can reduce pressure and frustration by providing individuals with multiple ways to communicate and express themselves, rather than solely relying on verbal communication (Mistry & Barnes, 2013; Sheehy & Budiyanto, 2014). It is evident that Makaton, because of its adaptability, can be an invaluable method to address diverse communication challenges and could be a fundamental resource for all professionals working with individuals with complex communication needs, including SLTs.

As professionals with specialised expertise in language and communication, SLTs play a central role in supporting individuals with complex communication needs. The AAC, including systems such as Makaton, forms a recognised part of SLTs’ scope of practice (Chua & Gorgon, 2019; Dada et al., 2017) and is often integrated into multidisciplinary intervention approaches (Kathard et al., 2022). Given its potential to enhance communicative access, SLTs’ proficiency with Makaton is critical to ensuring effective and inclusive service delivery.

Despite the recognised role of SLTs in AAC service delivery, limited research has examined their proficiency in using systems such as Makaton, particularly within low- and middle-income contexts such as South Africa (Dada et al., 2017; Pillay et al., 2020). In South Africa, most AAC research has focused on aided systems (Tönsing & Dada, 2016; Tönsing et al., 2019). For example, Tönsing and Dada (2016) explored teachers’ perceptions of using aided AAC to support expressive communication in special schools. Their findings revealed that while aided AAC was utilised, its implementation was hindered by challenges such as limited teacher training, resource constraints and inconsistent support from stakeholders. Tönsing et al. (2019) explored the impact of multilingualism on AAC use in South Africa, highlighting challenges faced by individuals in accessing AAC systems that support multiple languages. The study emphasised the need for culturally and linguistically inclusive AAC interventions to enhance communication effectiveness. Dada et al. (2017) focused on the overall implementation of AAC by South African SLTs. Findings revealed that SLTs’ approaches were often shaped by prior experience rather than structured, evidence-based methods, highlighting a need for more training and support in AAC implementation (Dada et al., 2017). Challenges in AAC implementation are not unique to South Africa, as evidenced by a United Kingdom study in which SLTs reported lacking confidence in their AAC-related roles (Norburn et al., 2016).

In South Africa, however, AAC implementation is further complicated by contextual realities. Many SLTs work in education and health sectors where high caseloads, resource constraints, and diverse linguistic and cultural populations present ongoing challenges (Pillay et al., 2020; Tönsing et al., 2019). In the absence of standardised guidelines, dedicated AAC policies and limited AAC-specific training available to SLTs in South Africa, therapists often rely on informal strategies rather than structured systems such as Makaton (Dada et al., 2017).

Makaton’s simplicity, affordability and flexibility make it an ideal tool for use in low-resource and multilingual environments. Its formal integration into SLT practice, however, appears to be limited (Faiz et al., 2022; Norburn et al., 2016). This is concerning, given its potential to enhance accessibility, support inclusion, and foster more effective communication in the South African context (Faiz et al., 2022). Makaton has been adapted to the South African context in terms of vocabulary, signs and symbols that are used to be more contextually appropriate and relevant to South Africa. As Makaton is an additional course offered outside of the undergraduate SLT degree, formal training by Makaton South Africa is required to use the approach effectively in clinical practice. Training can be completed by anyone who is interested including but not limited to SLTs, parents or doctors. Makaton training consists of six levels with level one being the first and six the highest level. Additionally, after completing level six, one can become a tutor but is not required (Makaton South Africa, n.d.). There is currently limited understanding of how Makaton is used in SLT practice in South Africa, the barriers therapists face, and where support is most needed. To address this gap, the current study explores South African SLTs’ perceived competencies and use of Makaton. In this study, *perceived competencies* refer to SLTs’ self-judged levels of knowledge, confidence and preparedness to use Makaton. This includes their understanding of the programme, the extent of their exposure and training, and their experiences applying it in practice. By examining and exploring their experiences, confidence levels and implementation practices, this research seeks to contribute towards strengthening AAC service delivery, particularly through tools that are inclusive, cost-effective and appropriate for South Africa’s diverse population.

### Main aim

To explore South African SLTs’ perceived competencies in and use of Makaton.

### Objectives

The objectives were as follows:

- To describe South African SLTs’ exposure to, understanding of, and experience with Makaton.
- To determine South African SLTs’ decision-making regarding when, and to whom, they recommend Makaton.

## Research methods and design

### Study design

A cross-sectional mixed-methods survey design was employed to collect both quantitative and qualitative data through an online questionnaire (Brink et al., 2018; Leedy & Ormrod, 2020).

### Study population and sampling strategy

South African SLTs who have active Health Professions Council of South Africa (HPCSA) registration, are currently practising in South Africa, and have completed at least level 1 Makaton training. The final sample consisted of 57 participants. A total of 1095 SLTs registered with HPCSA in January 2018: 1095 (Pillay et al., 2020), and a total of 1574 individuals completed Makaton training through Makaton South Africa. It is estimated that approximately 65% of this group are SLTs (Makaton South Africa, n.d.).

Purposive sampling was used for recruitment (Brink et al., 2018). Recruitment was conducted through Makaton South Africa, the South African Speech-Language-Hearing Association (SASLHA) and social media platforms, including Facebook groups such as Allied Health in South Africa and South African Audiologists and Speech-Language Therapists. Participation was voluntary, with eligible individuals providing informed consent before completing the online survey.

Of the 57 participants, nearly half were based in Gauteng (47.4%), followed by approximately one-quarter from the Western Cape (24.6%). The vast majority of participants (87.7%) worked in urban settings, with the majority employed in private preschools. The paediatric population was the most commonly served, although several participants also reported working with adolescents and adults. Almost half of the participants had between 1 and 5 years of professional experience, suggesting a predominantly early-career sample. Table 1 presents a summary of the participants’ demographic and professional characteristics.

**TABLE 1:** Demographic and professional characteristics of participants (*N* = 57).

Demographics	*n*	%
**Geographic location**		
Gauteng	27	47.4
Western Cape	14	24.6
KwaZulu-Natal	6	10.5
Mpumalanga	3	5.3
North West	2	3.5
Easter Cape	2	3.5
Free State	1	1.8
Limpopo	1	1.8
Northern Cape	1	1.8
**Setting**		
Urban	50	87.7
Rural	7	12.3
**Employment setting**		
Public health sector	8	14
Private health sector	21	36.8
Preschool (Public)	3	5.3
Preschool (Private)	23	40.4
Primary school (Public)	7	12.3
Primary school (Private)	15	26.3
Secondary school and/or High school (Public)		
Secondary school and/or High school (Private)	2	3.5
Schools for Learners with Special Educational Needs (LSEN) (Public)	21	36.8
Schools for Learners with Special Educational Needs (LSEN) (Private)	15	26.3
Institution for higher education (e.g. a university)	5	8.8
**Population participants work with**		
Paediatric	56	98.2
Adolescent	28	49.1
Adults	19	33.3
Geriatric	13	22.8
**Years of experience**		
1–5	27	47.3
6–10	13	22.8
11–15	6	10.5
16–20	6	10.5
21–25	1	1.8
26–30	3	5.3
31–35	1	1.8

Note: Employment setting and population served allowed multiple selections.

### Data collection method and tool and/or instrument

The survey was administered online via Qualtrics. The questionnaire was developed by incorporating and adapting questions from existing surveys (Barman et al., 2023; Goldbart et al., 2014; Moorcroft et al., 2019; Ward et al., 2023). The survey took approximately 15 min to complete. Data collected included demographic information, participants’ understanding of Makaton, and their perceived competencies, as well as the utilisation of Makaton. Weekly reminders and follow-up posts were shared via the approved platforms to encourage participation.

### Content validity and reliability

The questionnaire’s validity was established through expert review by two qualified SLTs and a statistician (triangulation), a pilot study, and alignment with established survey principles. Reliability was assessed via internal consistency, using McDonald’s omega (equals 0.754) as an alternative to Cronbach’s alpha (Brink et al., 2018; Hayes & Coutts, 2020; Heale & Twycross, 2015). Makaton usage and perceived effectiveness were measured using 4-point Likert-scale items. For the qualitative component, trustworthiness, credibility, transferability, dependability and confirmability were ensured through in-depth analysis, peer review and clear documentation of the research process (Leedy & Ormrod, 2020).

### Data analysis

Quantitative data were analysed using descriptive and inferential statistics in Statistical Package for the Social Sciences (SPSS) (Version 29). Descriptive statistics included frequencies, percentages, means (M), medians (Mdn), standard deviations (s.d.) and interquartile ranges (IQR). The Wilcoxon signed-rank (WSR) test was used for comparisons involving ordinal Likert-scale data (Brink et al., 2018). Qualitative data were analysed thematically using Braun and Clarke’s six-phase framework (Braun & Clarke, 2021a, 2021b; Vaismoradi et al., 2013).

### Ethical considerations

Ethical clearance to conduct this study was obtained from the University of Pretoria Faculty of Humanities Research Ethics Committee on 03 November 2024. The ethics approval number is 16188935 (HUM007/0824).

## Results

The two sections, ‘Understanding Makaton’ and ‘Perceived competences’, combined aimed to provide information on SLTs exposure, understanding and experiences with Makaton to be able to provide information on the perceptions SLTs have with Makaton.

### Understanding Makaton

The study included 57 participants (*N* = 57). The majority (*n* = 56; 98.2%) correctly identified the definition of Makaton. The same proportion (*n* = 56; 98.2%) indicated that ‘anyone interested’ could attend formal Makaton training. Occupational therapists, SLTs, parents and/or caregivers and teachers were each selected by 66.7% (*n* = 38) of participants as suitable for training, while doctors and other professionals were least frequently selected.

Regarding familiarity with Makaton, 50.9% (*n* = 29) of participants reported being ‘moderately acquainted’, 26.3% (*n* = 15) ‘very acquainted’, 14.0% (*n* = 8) ‘neutral’ and 8.8% (*n* = 5) ‘slightly acquainted’. While most of the participants had between 1 year and 5 years of professional experience, participants’ years of experience did not appear to be a determining factor in how well they were acquainted with Makaton. Some experienced SLTs reported feeling only ‘slightly acquainted’, while some early-career SLTs reported high levels of familiarity.

### Perceived competencies of Makaton

**FIGURE 1:**
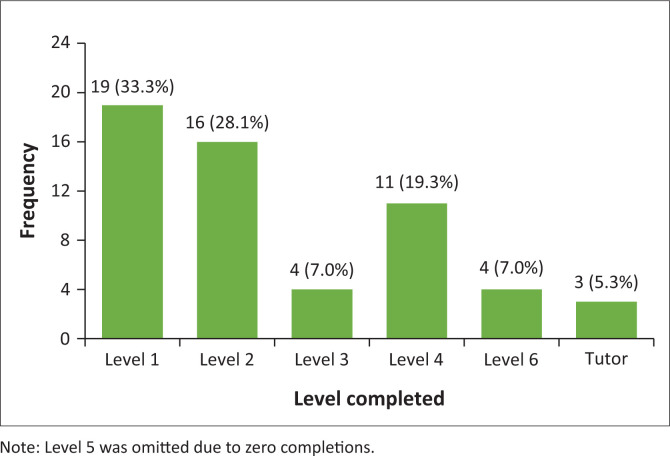
Highest levels of Makaton training completed.

There are six levels of Makaton training, with ‘tutor’ being an optional advanced level. As shown in Figure 1, the majority of participants had completed Level 1 or 2.

Slightly over half of the participants (*n* = 30; 52.6%) indicated they would like to complete further Makaton training. Reasons for not progressing to higher levels included financial constraints (*n* = 16; 28.1%), limited opportunities (*n* = 13; 22.8%) and time constraints (*n* = 12; 21.1%). A few participants (*n* = 4; 7.0%) had completed all levels except the tutor level, while three (5.3%) were not interested in pursuing further training. Several participants selected ‘other’ (*n* = 8; 14.0%), with explanations including a preference for alternative systems such as South African Sign Language (SASL) and satisfaction that the content of Levels 1 and 2 addressed their needs.

The majority of participants (*n* = 37; 64.9%) first learned about Makaton during their undergraduate studies. Other sources of exposure included social media (*n* = 17; 29.8%), colleagues or workplace settings (*n* = 16; 28.1%) and friends or family (*n* = 8; 14.0%). More than half of the sample (*n* = 31; 54.4%) reported that Makaton was ‘very effective’ in supporting communication. Only one participant (1.8%) indicated that it was ‘not effective’. To gain a better understanding of the perceived value of Makaton, participants were asked to list reasons they found it beneficial. Four key themes emerged from their responses: (1) multimodal communication, (2) accessibility and practicality, (3) speech and language development and (4) inclusion and social use. These are summarised in Table 2.

**TABLE 2:** Speech-language therapists’ views on Makaton’s value for use in South Africa.

Theme	Example response
**Multimodal communication:** Makaton is a multimodal form of communication that provides support through visual and verbal forms to support communication.	‘Multimodal – Uses signs with speech to communicate’. (P3, Urban, Mpumalanga)
**Accessibility and practicality:** No additional devices are required, and Makaton is flexible, easy to learn and implement.	‘It is a low-cost option for lower socioeconomic areas and populations who may not be able to afford high-tech AAC’. (P23, Rural, Public Health Sector)
**Speech and language development:** Participants indicated that Makaton encourages spoken language by making use of signs to support this development. Makaton can also play an important role in early communication intervention. Participants explained that Makaton can support and potentially act as a bridge for literacy.	‘It promotes Speech skills while using gestures as building blocks’. (P10, Primary school [Private], Paediatric)
**Inclusion and social use:** Makaton can support an inclusive environment as Makaton can be used within a variety of populations and in different settings, for example, home and school. Makaton can also provide a form of communication that can help people with communication difficulties to interact with the world around them and lessen frustration as they are able to express themselves.	‘Empowerment of all parties who make use of it. Inclusion of those who do not communicate orally’. (P39, Private Health Sector, Paediaric)

AAC, augmentative and alternative communication.

To gain a better understanding of the challenges South African SLTs experience when implementing Makaton, participants were asked to describe difficulties they had encountered with implementing Makaton. Five common themes emerged from their responses: (1) lack of awareness and training, (2) financial and resource constraints, (3) consistency and carryover, (4) motor and cognitive challenges and (5) cultural and regional limitations. These themes are summarised in Table 3.

**TABLE 3:** Speech-language therapists’ challenges with implementing Makaton in South Africa.

Theme	Example response
**A lack of awareness and training:** Makaton is not widely known by parents and in public settings. There is a lack of knowledge about Makaton within South Africa, and Makaton can be seen as sign language rather than as a form of AAC.	‘Makaton is not widely known by the general population’. (P52, Urban, Gauteng) ‘Lack of understanding of benefits is seen as sign language and not as part of AAC’. (P37, Urban, Western Cape)
**Financial and resource constraints:** Training and resources are costly and resources are not easily accessible.	‘Cost of the training’. (P41, Western Cape, LSEN Public) ‘Not taught to parents for the lowest cost possible when they need it most’. (P33, Gauteng, Urban)
**Consistency and carryover:** There is limited carryover from the therapy session to other settings, for example, school and home. Signs can also vary between regions, causing inconsistencies with implementation.	‘Carry over in the home environment if parents are not 100% on board’. (P27, Mpumalanga, Preschool Private)
**Motor and cognitive challenges:** Motor and cognitive abilities can complicate the implementation of Makaton, as signs might be too complex for the population with motor difficulties to produce. The population might also require additional practice to use the signs effectively.	‘Most children have fine motor skills difficulties and find it difficult to execute the movements required to make the signs and it is limiting in that regard’. (P6, Gauteng, Urban)
**Cultural and regional limitations:** Makaton was not developed in South Africa, which might lead to some signs not being culturally appropriate. In some cultures, it might be believed that signs might hinder speech development, leading to the stigma and/or myths around Makaton.	‘Caregivers’ and educators’ misperception/myth that it will inhibit speech! The myth that it is only for non-English speaking learners’. (P24, Western Cape, Urban)

AAC, augmentative and alternative communication; LSEN, learners with special educational needs.

### Using Makaton

Participants reported on both the frequency of their Makaton use and their confidence in using Makaton based on a 5-point Likert scale. The most selected frequency responses were ‘often’ (*n* = 24; 42.1%) and ‘very often’ (*n* = 19; 33.3%); while only two participants (3.5%) indicated that they never use Makaton. In terms of confidence, only five participants (5.5%) indicated that they feel ‘very confident’ using Makaton.

Open-ended responses indicated that the participants use Makaton with a wide range of populations. These included individuals with ASD, developmental delay (DD), global developmental delay (GDD), cerebral palsy (CP), Down syndrome, attention deficit hyperactivity disorder (ADHD), foetal alcohol syndrome, apraxia of speech (AOS) and motor speech disorder, selective mutism, language disorder and delays including specific language impairments and late talkers, severe intellectual disability (SID), hearing impairment and children who are deaf. They also reported using it with children receiving early communication intervention (ECI) and with adults with aphasia and traumatic brain injury (TBI). Additional populations mentioned included those with genetic conditions such as Angelman syndrome, Rett syndrome, Velocardiofacial syndrome (VCFS), Dravet syndrome and Kagami-Ogata syndrome.

**FIGURE 2:**
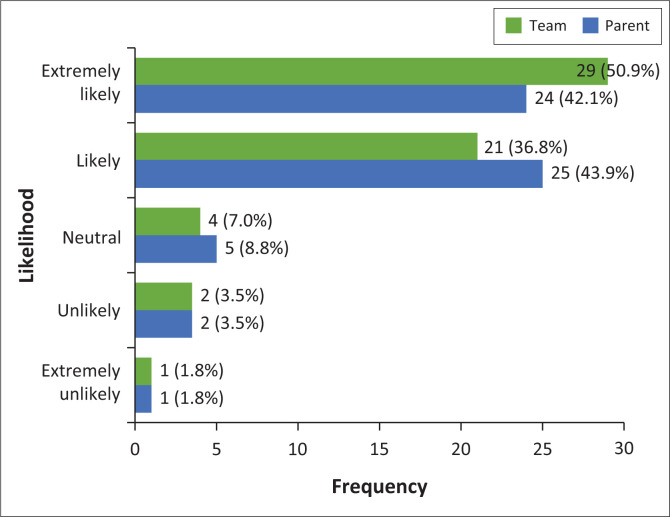
Likelihood of recommending Makaton to parents and team members.

Figure 2 presents participants’ ratings of how likely they are to recommend Makaton to parents and therapy team members, based on a 5-point Likert scale (1 = ‘extremely unlikely’ to 5 = ‘extremely likely’). Participants were highly likely to recommend Makaton to both parents (M = 4.21, Mdn = 4.00, s.d. = 0.88, IQR = 1.00) and team members (M = 4.32, Mdn = 5.00, s.d. = 0.89, IQR = 1.00). The WSR test showed no statistically significant difference between the two groups (WSR = −1.386, *p* = 0.166), indicating similar levels of endorsement.

Participants who indicated they were ‘extremely unlikely’ or ‘unlikely’ to make the recommendation to both parents and the therapy team noted a preference for more formal communication systems, such as SASL or alternative AAC methods.

Table 4 summarises the main themes identified for recommending Makaton to parents and team members.

**TABLE 4:** Reasons for recommending Makaton to parents and team members.

Theme	Example response
**Parents**	
**Supports and enhances communication:** Makaton can support language development and provide an accessible form of communication.	‘Makakon can be used to enhance speech and language and it can be used to target reading and literacy skills for children with severe intellectual disabilities’. (P6, LSEN Public, Paediatric)
**Support for parents and caregivers:** Makaton offers support for parents and caregivers, as it is easy to learn and implement.	‘In order to support the parents in feeling more confident when communicating with their children’. (P50, LSEN Private, Paediatric)
**Accessibility:** Makaton is cost-effective, requires no additional resources, and can be implemented across settings and routines to support carry-over.	‘Easy to access for the parents [*no resources needed*], immediate relief for patients who cannot make needs known’. (P35, Western Cape, Urban)
**Social benefits:** It provides a way to express wants and needs, which can reduce frustration and support positive interactions.	‘It would be helpful for carryover purposes for daily activities and socialising’. (P13, Eastern Cape, Rural)
**Team members**	
**Multidisciplinary use and consistency:** Makaton can be used by a variety of professionals, supporting communication across settings and promoting consistency and carry-over.	‘There will be greater opportunities and success for children to use Makaton if the whole team is involved’. (P9, LSEN Private, Paediatric)
**Practical:** Makaton is easy to learn and implement across therapy environments.	‘Easy total communication between clinicians and patients as well as caregivers’. (P13, Public Heath Sector, Adult)
**Support for therapy goals and outcomes:** Makaton helps teams reach therapy goals and understand communication attempts.	‘Lessens workload in the long run – client understanding and interaction contributes to successful therapy outcomes’. (P39, Mpumalanga, Rural)
**Support for inclusion and collaboration:** Integration across disciplines promotes inclusive environments and empowers teams to deliver holistic care.	‘Having Makaton training can empower the team members especially when having a client that is seen within a multidisciplinary team and everyone have a way to support and improve communication’. (P56, Private Health Sector, Paediatric)

LSEN, learners with special educational needs.

Finally, participants were asked for suggestions to improve the training and implementation of Makaton in South Africa. The following key themes emerged: (1) accessibility and affordability, (2) training and development opportunities, (3) awareness and integration, (4) contexts and practical application (see Table 4). A brief explanation of each theme is provided in Table 5.

**TABLE 5:** Suggestions for improving the training and implementation of Makaton in South Africa.

Theme	Example response
**Accessibility and affordability:** Participants indicate that reducing the cost of training and available resources, as well as having more training opportunities, especially online. Potentially getting additional funds for training from the government to make Makaton more accessible.	‘The government or education department should make Makaton training compulsory for all allied health professionals and LSEN educators and the government should fund the training’. (P15, LSEN Public, Adolescent)
**Training and development opportunities:** Providing more level five, six and tutor training. Providing opportunities, for example, refresher courses to support continuous professional development (CPD) points.	‘More opportunities to do level 5/6 and more opportunities to do the tutor training’. (P5, Western Cape, Urban)
**Awareness and integration:** Participants indicated that Makaton could be integrated into undergraduate studies for healthcare professionals. In addition, more marketing, for example, through social media or at schools.	‘Incorporate basic levels of Makaton into University courses, particularly in healthcare’. (P51, Gauteng, Urban) ‘Making Makaton more widely used. e.g. putting signs up in shopping centres and other public areas. So that it becomes more well-known and that more people are exposed to Makaton’. (P32, Gauteng, Urban)
**Contexts and practical application:** Integrate Makaton into different settings, for example, by placing signs in shopping centres in order for more exposure and integration. Contexts and practical implementation: Participants indicated that Makaton training could be implemented in specific settings, for example, schools. Expanding vocabulary, for example, theme-related vocabulary for schools. Lastly, to provide more resources, for example, handouts for parents.	‘Training could be more tailored for a school environment (topics and vocab that are likely frequently used)’. (P38, LSEN Private, Paediatric) ‘Maybe more specific guidelines for parents/handouts’. (P48, Mpumalanga, Urban)

LSEN, learners with special educational needs.

## Discussion

There has been a notable lack of research investigating the perspectives and use of Makaton among South African SLTs. Previous research focused on aided AAC (Tönsing & Dada, 2016; Tönsing et al., 2019) with limited information about SLT’s perception of Makaton. Overall, the findings suggest a positive response to Makaton as a form of AAC. Participants showed a clear understanding of what Makaton is and recognised its value in supporting communication, despite reporting several challenges related to its implementation.

Research conducted in South Africa on the distribution of the SLT workforce indicates that the majority of SLTs are located in Gauteng and the Western Cape (Pillay et al., 2020). Similarly, in this study, more than half of the responses came from these provinces, with the majority of participants working in urban settings. Many were employed in private sector contexts, such as private preschools, while those in the public sector were often based at Learners with Special Educational Needs (LSEN) schools. This aligns with broader trends of SLTs working across diverse clinical and educational settings (Agaronnik et al., 2019; Berenguer et al., 2022; Biggs et al., 2018; Blackstone et al., 2021; Dada et al., 2017; Sigafoos & Gevarter, 2019). This context is important when interpreting the findings of this study, as SLTs working in urban, better-resourced, and private settings may have greater exposure to or access to training in tools such as Makaton. As such, the results may not fully reflect the challenges faced by SLTs in rural or under-resourced environments, highlighting the need for future research in those settings.

Although participants had varying years of professional experience, familiarity with Makaton did not seem to follow a clear pattern based on experience alone. This suggests that professional experience may not be the strongest predictor of AAC competence. Instead, as previous studies have found, SLTs’ use of AAC tools, such as Makaton, is often shaped by individual exposure, access to training, and clinical context rather than by structured, evidence-based preparation (Chua & Gorgon, 2019; Dada et al., 2017).

An important finding from this study is that participants perceived Makaton as an effective tool for supporting communication. However, more than half reported feeling only ‘moderately acquainted’ with it, and most had completed only Level 1 or 2 training. While higher-level training was limited, participants commonly cited lack of access rather than lack of interest or perceived value as the main barrier. This reflects similar findings in previous South African research, where SLTs identified limited AAC training opportunities as a challenge to service delivery (Dada et al., 2017). The fact that participants expressed strong interest in further training, despite limited access, underscores the profession’s readiness to engage more deeply with AAC. This interest, however, contrasts with the lack of accessible pathways to develop competence, pointing to a structural gap in professional preparation. Addressing this gap requires more than isolated training opportunities; it calls for a national framework that embeds AAC within curricula, ensures equitable access across provinces, and provides sustainable professional development routes. In this way, the findings highlight both the urgency and the opportunity for expanded, structured training, echoing broader international debates on AAC training equity (Fayyaz et al., 2024). It also adds to the growing body of evidence from South Africa (Dada et al., 2017) calling for improved national guidelines and training pathways to support SLTs in delivering AAC services to vulnerable populations.

This study also explored South African SLTs’ perceptions of Makaton, focusing on its effectiveness and the challenges of implementation. Participants viewed Makaton as an effective AAC method, echoing international research that demonstrates the value of AAC systems in enabling individuals with complex communication needs to express themselves (Faiz et al., 2022; Mistry & Barnes, 2013; Sheehy & Budiyanto, 2014). Importantly, the reasons cited for Makaton’s effectiveness – its multimodality, versatility across contexts, and ability to support social inclusion – suggest that SLTs see it as a flexible tool that can be integrated into diverse clinical and educational settings. In addition, participants reported using Makaton with a wide range of populations, reinforcing its adaptability for multiple conditions and aligning with its design as a multimodal, accessible communication approach (Bednarski, 2016; Brignell et al., 2018; Faiz et al., 2022). This broad application strengthens the argument that Makaton is not only comparable to other AAC approaches but particularly well-suited for low-resource and multilingual environments such as South Africa, where simplicity, affordability and flexibility are essential. These findings therefore align with broader AAC literature emphasising participation across environments (Handberg & Voss, 2018; McNaughton et al., 2019; Waddington et al., 2017), while also highlighting the unique potential of Makaton to address systemic barriers in the South African context.

Participants identified several challenges with the implementation of Makaton, including a lack of awareness and training, resource and time constraints, difficulties with consistency and carryover, and cultural and regional barriers. These challenges were consistent with findings from both South African and international research (Dada et al., 2017; Norburn et al., 2016; Tönsing & Dada, 2016; Tönsing et al., 2019). These challenges point to the need for improved awareness campaigns, greater access to affordable training, and the development of culturally responsive resources that support consistency and carryover across South Africa’s diverse setting. The fact that SLTs continued to recommend Makaton to parents and team members, and expressed confidence about who could be trained, suggests a shift from relying primarily on informal AAC strategies (as previously reported by Dada et al., 2017) towards recognising the value of structured systems like Makaton. This reflects growing professional openness to integrating formal AAC into service delivery, even in resource-constrained contexts and strengthens the case for national strategies that prioritise equitable AAC training and culturally relevant implementation guidelines (Dada et al., 2017; Norburn et al., 2016; Tönsing & Dada, 2016; Tönsing et al., 2019).

Participants offered practical suggestions for improving Makaton training and implementation. These suggestions point to a broader need for more accessible and contextually relevant training in South Africa. The emphasis on embedding Makaton in daily routines and across environments reflects a growing recognition of the importance of ecological and inclusive approaches to AAC intervention as suggested by Tönsing et al. (2019) as well as Dada et al. (2017). These insights reinforce calls for stronger integration of AAC, including Makaton, into both pre-service education and ongoing professional development. Importantly, they highlight the value of moving beyond clinic-based training models towards more functional, community-based applications that support real-world carryover and inclusion.

### Strengths and limitations

The strengths of this research include that it provides valuable insight into SLTs’ perceived competencies and use of Makaton, an area that has received minimal attention in South African research and remains largely absent from international literature. To the best of the researcher’s knowledge, this is the first study in South Africa to specifically explore SLTs’ experiences and perceptions of using Makaton as a form of AAC. This contribution is particularly important given the limited research on AAC in low- and middle-income countries (Dada et al., 2017; Pillay et al., 2020) and adds to the body of literature from underrepresented contexts.

The relatively small sample size limits the generalisability of the findings. Most responses came from only two provinces and therefore reflect the perspectives of only a portion of the broader SLT population. The use of self-report data introduces the possibility of response bias. Furthermore, the cross-sectional nature of the study means that no causal relationships can be inferred. The use of non-probability sampling further limits the generalisability of the findings, as participants were selected based on inclusion criteria and voluntary response.

### Implications or recommendations

This study highlights the need for increased training opportunities and structured support to enhance SLTs’ confidence and consistent use of Makaton in clinical practice. Given the limited research on Makaton within the South African context, especially in rural or under-resourced settings, further studies are needed to explore implementation across a broader and more diverse sample. A larger-scale replication of this study would provide a more comprehensive understanding of SLTs’ experiences and competencies nationally. In addition, future research could explore comparative studies between Makaton and other AAC systems, such as Tiny Handz (basic South African sign language for hearing babies and toddlers), to assess relative usability, accessibility, and effectiveness in supporting communication. Further investigation into the impact of Makaton on client outcomes and the role of workplace training and support structures would also be valuable.

## Conclusion

This study provides valuable insight into how South African SLTs perceive and use Makaton as an AAC method. It highlights Makaton’s relevance in supporting individuals with complex communication needs and raises awareness of its use within the South African context. The qualitative data offered a deeper understanding of both the value SLTs associate with Makaton and the challenges they face in implementing it effectively.

Participants also shared practical suggestions for improving training, access and integration. These findings can inform the development of locally relevant AAC guidelines and support the inclusion of Makaton in undergraduate curricula. While the sample was limited, the study contributes to a growing evidence base and emphasises the need for further research to support AAC implementation in diverse South African settings.

The results from this study may help improve SLTs’ confidence, consistency, and effectiveness in using AAC strategies in diverse clinical settings. These changes have the potential to enhance South African SLTs’ engagement with Makaton as a tool to support communication (Bednarski, 2016; Faiz et al., 2022).
